# COVID-19 and Nursing: for a commitment to Health Education

**DOI:** 10.1590/1518-8345.0000.3473

**Published:** 2021-08-02

**Authors:** Ethel Maciel

**Affiliations:** 1 Universidade Federal do Espirito Santo, Departamento de Enfermagem, Vitória, ES, Brazil.; 2 Coordenadora do Laboratório de Epidemiologia da Universidade Federal do Espirito Santo, Vitória, ES, Brazil.; 3 Presidente da Rede Brasileira de Pesquisa em Tuberculose (REDE-TB), Rio de Janeiro, RJ, Brazil.

**Figure:**
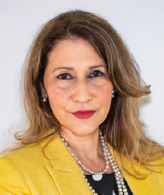

The year 2020 proved that the evolution of science in the last century has transformed the threats caused by microorganisms into a challenge that can be overcome. In the last pandemic that devastated the planet, the influenza pandemic in 1918, the best scientists in the world were unable to identify the virus, many measures adopted were insufficient, and attempts to develop an effective vaccine proved useless. In this COVID-19 pandemic, a year after the first cases were identified in China, the sharing of scientific information, the improvement of technologies, and the correct use of scientific methods gave us the first vaccines against COVID-19 and the possibility of controlling the disease within a period never seen in history.

However, it is not enough that, as a society, we have developed safe and effective vaccines. They must reach all people. This is where our most significant challenges are concentrated. We have never had so much information available and, at the same time, never before has ignorance been so widespread. Articulated groups disclose disinformation and generate distrust and fear regarding the only weapons to prevent the disease. The role of Nursing as health educators is, today, paramount.

The data presented in the report on the situation of Nursing in the World show that Nursing is the largest occupational group in the health sector, accounting for approximately 59% of health professions^1^. Furthermore, by being part of the Family Health teams in Brazil, very close to the communities, they can have a dialogue that is based on an accessible and contextualized language with the reality of each individual, and that is essential for the promotion of health and disease prevention in this moment of the pandemic, combating misinformation^2^.

Unfortunately, we witness that the control strategies of COVID-19 in Brazil were guided by expanding hospital beds, focusing on an assistance action, and little focused on Primary Health Care (PHC). Nursing in Brazil can do much more, focusing its work on PHC actions in intensified home visits and emphasizing the care of primary diseases, such as diabetes and hypertension control and monitoring cases of people with COVID-19 and their contacts. The use of flowcharts in PHC when assessing a suspected case of COVID-19 has the potential to organize access to care and prevent complications from the disease^3^.

This organization is even more imperative concerning vaccination. Nursing is ahead of all 38 thousand vaccination rooms in Brazil and can lead health education actions while still not having a mass vaccination for our population. This health education action could greatly impact the population’s adherence to vaccines and combined with prevention and health promotion actions during the pandemic, to give visibility to nursing work in Brazil^4^^-^ 5. There is much to be done. Nursing is prepared to assume its role in the history that is being written during the COVID-19 pandemic.
